# Predictors of Unmet Healthcare Needs during Economic and Health Crisis in Greece

**DOI:** 10.3390/ijerph20196840

**Published:** 2023-09-27

**Authors:** George Pierrakos, Aspasia Goula, Dimitra Latsou

**Affiliations:** 1 Department of Business Administration, School of Administrative, Economics and Social Sciences, University of West Attica, 12243 Athens, Greece; gpierrakos@uniwa.gr (G.P.); agoula@uniwa.gr (A.G.); 2 Department of Economics and Business, School of Economics, Business and Computer Sciences, Neapolis University Pafos, Pafos 8042, Cyprus

**Keywords:** healthcare, unmet healthcare needs, economic crisis, health crisis, Greece

## Abstract

(1) Background: The aim of this study was to identify predictors of the unmet healthcare needs during the financial and recent health crisis in Greece. (2) Methods: Time series analysis was performed for the years 2008 through 2022 using the Eurostat database. The dependent variable was the percentage of people who reported unmet need for medical care. Demographic, socioeconomic, and health data, as well as health expenditures, were used as independent variables. Correlation analysis and simple linear regression models were conducted to analyze the results. (3) Results: Unmet health needs in Greece increased from the start of the crisis until 2016, as a gradual de-escalation of the crisis was observed. However, in 2019 the country recorded the second highest level of unmet needs for medical care before the health crisis. Limitations in usual activities, reporting bad/very bad health status, being unemployed, and having low income increased the likelihood of unmet needs. Health expenditures (public or private) were also significant determinants of unmet healthcare needs. (4) Conclusions: The increased unmet health needs widen inequalities in health and healthcare access. Therefore, health policies should eliminate barriers which restrict the access to health and enhance healthcare services, developing conditions for citizens’ well-being.

## 1. Introduction

Social needs include the concept of health requirements; however, these needs cannot be identified objectively, as they are either the subjective requirements of every person or the collective needs of society that emerge on the basis of objective standards of prioritization and assessment. The coverage of social requirements is balanced between the necessity for the long-term viability of social systems and the social costs, resulting from the non-satisfaction of social demands and taking into consideration the restrictions of the resources and budget [1,2].

Bradshaw divided needs into several different categories after taking these limitations and assessment challenges into account: “normative need” as “desired standard” or “objective”; “felt need” as synonymous with desire; “expressed need” associated with demand [3]. Moreover, according to the European Union (EU), unmet health needs are defined as the percentage of people that require healthcare but have reported a delay in receiving it in the 12 months prior to the incident. The EU claims that the following elements are contributing to unmet health needs: (a) high costs for services; (b) geographic distance and transportation issues; (c) slow system responses and waiting lists. The limitations on access to health services offered by the State include all of the above factors. However, the demand for healthcare persists and does not go away, which has the effect of either manifesting later as a major sickness or as forcing the person to pay for it privately [4]. The examination and the evaluation of health needs are important as they constitute the basis for figuring out understanding how health services are offered, and they basically constitute market research for the development of services as a public benefit that will be accessible to all individuals without social exclusion [5].

The health systems in Europe were greatly strained by the pandemic, with resources being redirected to manage the crisis. As a result, many health services were impacted during the early stages of the pandemic and during its most severe periods [6]. People were advised to stay at home to prevent the spread of the virus and delayed or rescheduled their appointments in health services. Thus, this disruption of health services led to an increase in unmet healthcare needs.

Most of the population in EU countries reported that they had no unmet medical care needs for financial reasons, geographic reasons, or waiting times in 2018 or 2019, according to the EU-SILC [7]. The extent of unmet healthcare needs was dramatic, although it differed across various types of care during the pandemic. Approximately 20% of people across EU countries highlighted forgone medical care during the first wave of the pandemic, and nearly one-fifth of respondents (18%) reported having a medical issue for which they had not yet received examination or treatment in spring 2022. Regarding access to hospital or specialist care, half of the EU population in 2020 and more than two-fifths in 2021 reported obstacles. Overall, unmet medical care needs remained high in 2021 and 2022 [8].

As far as dental care is concerned, during the pandemic, there was a disturbance in obtaining dental care, and more than 25% people in the EU experienced unmet needs in 2021 and 2022. An explanation may be that dental care is not fully covered in public health schemes in several countries, necessitating out-of-pocket expenses or private health insurance [9].

The pandemic has led to growing unmet needs for mental healthcare, even though there was a rapid adaptation to new service formats such as online therapy and several measures which were taken by EU countries to increase mental health support. The percentage of European adults who reported some unmet needs for mental care increased from 20% in 2021 to 23% in 2022 [8]. Worries arise as 53% young Europeans reported unmet needs for mental healthcare in 2021, with a slight decrease in 2022 (49%). However, the situation was exacerbated during the pandemic, as a large proportion of people seeking mental healthcare reported difficulties obtaining it in the previous years [10].

Greece presents a unique setting in which to analyze inequalities in access to healthcare services. The repercussions of financial and recent health crises have been a challenge for the country to handle [11]. GDP per capita was estimated at EUR 18,310 in 2011, decreased to EUR 16,270 in 2014, and increased to EUR 19,670 in 2022 [12]. Greece’s healthcare system is mostly focused on hospitals. As a consequence, those in need of healthcare will mainly seek the availability of services at hospitals’ outpatient clinics that provide primary healthcare. Public health expenditures are one area being contained as a part of fiscal sustainability measures. More specifically, public expenditures were estimated at EUR 12.3 billion in 2011, which decreased to EUR 8.1 billion in 2014 and reached EUR 9.7 billion in 2020 (latest data) due to the needs of the pandemic [13]. A steady increase in public health expenditures has been observed since 2015. Private expenditures were estimated at EUR 6.4 billion in 2011, which decreased slightly to EUR 5.9 billion in 2014 and remained at approximately EUR 6 billion in 2020 [13]. Private healthcare expenditures are still expensive, up to 30% over the average across Europe. This cost is mostly for medication on prescription and primary healthcare.

Several predictors of the unmet healthcare needs have been mentioned by the international literature. More specifically, females [14], younger people [14], people with chronic conditions [15], and people with bad health status [16] have been shown to be more likely to self-report unmet healthcare needs. Empirical evidence indicates that people with low income have greater healthcare needs [17,18,19]. Additionally, several studies have provided significant evidence regarding economic activity status-related inequalities in unmet healthcare needs. Unemployed or self-employed people were more likely to report unmet healthcare needs in comparison with employees [20,21]. Lastly, political circumstances and economic crises were shown to affect the unmet healthcare needs of a population [22,23].

The purpose of this research was to present the unmet medical needs during the financial and recent health crises in Greece by income quintile, region (NUTS 2), and occupational status compared with European Union 27, and to identify predictors of the unmet healthcare needs within the population of the country.

## 2. Materials and Methods

This research was a cross-sectional study and represented a secondary analysis of data obtained from the Eurostat database [24]. The analysis used time series from 2008 to 2022, and several indicators were processed, as shown in Table 1.

### Statistical Analysis

Descriptive statistics (percentages) were used to present the differences in unmet medical needs in various categories, including Greece, the European Union 27 (EU27), region, income quintile, and occupational status. Additionally, a correlation analysis was conducted using the Pearson correlation coefficient in order to investigate the directional relationship between the unmet medical needs and the examined indicators. Only eight indicators showed a correlation with unmet medical needs, which were used for further analysis to explore the effect size. For this reason, eight simple linear regression models were performed. More specifically, in each model, the unmet medical needs were inserted as a dependent variable and the examined indicators were separately inserted as independent variables. The results were considered statistically significant when the *p*-value was ≤0.05. The analysis was performed using the Statistical Package for Social Sciences (SPSS) version 25.

## 3. Results

### 3.1. Unmet Medical Health Needs in Greece versus EU27

**Figure 1:**
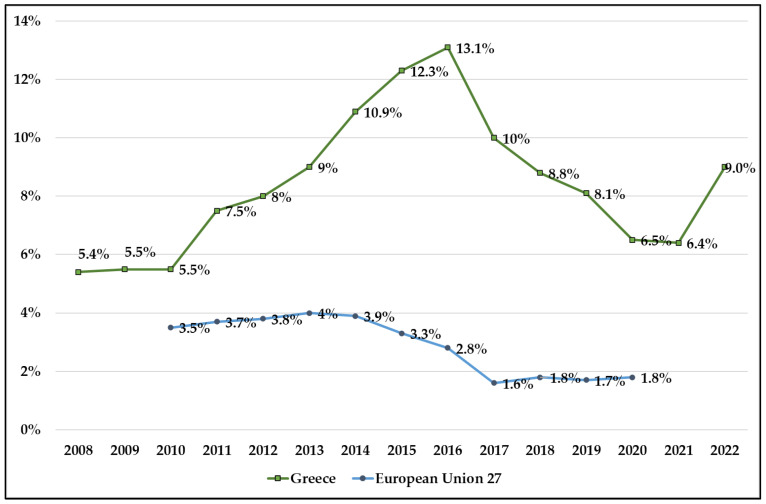
Unmet health needs for medical care in Greece and EU27. Source: Eurostat: 2022 (HLTH_SILC_08).

EU Member States increased access to healthcare significantly between 2005 and 2009. Specifically, the number of those indicating an unmet need for healthcare due to cost, travel distance, or length of wait declined steadily from 24 million in 2005 to 15 million in 2009. This increasing tendency has changed since 2009, showing an evident indication of the destruction caused by the financial and economic crisis. In 2013, 18.6 million individuals (4% of the EU’s population) expressed unmet healthcare needs. Regarding Greece’s unmet health needs in 2010, there was little distinction compared to those of the EU27; however, during the country’s economic crisis, the gap sharply grew from 2011 to 2016. Greece exhibited triple the total number of inadequate needs compared to the EU27 nations. The proportions in Greece have been gradually declining since 2017, although they remain very high when compared to the other EU27 countries (Figure 1).

According to Eurostat (2021) [25], the main reasons given for unmet health needs in EU27 are long waiting list (19.4%), followed by financial reasons (13%) and distance or transportation (4%). The Greek data do not differ: financial reasons (14.4%), waiting list (12.5%), and distance or transportation (5%).

### 3.2. Unmet Dental Health Needs in Greece vs. EU27

**Figure 2:**
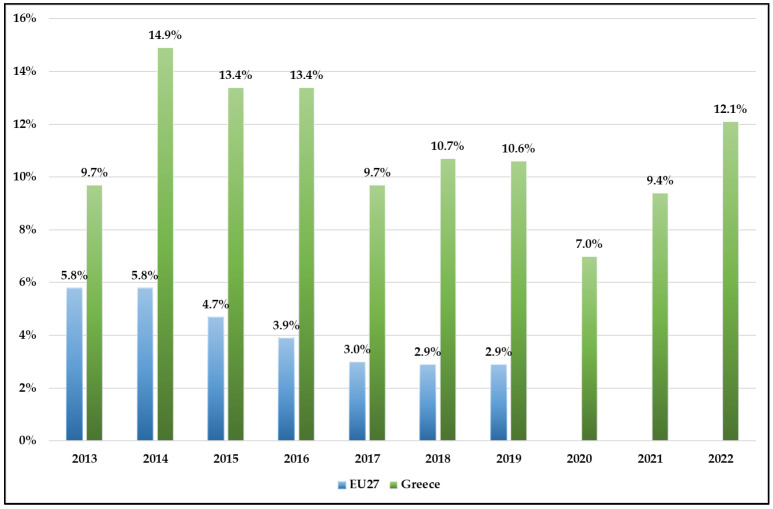
Unmet dental health needs in Greece and EU27. Source: Eurostat 2022 (HLTH_SILC_09).

Until the year 2015, about 5% of the EU27 population experienced unmet dental care needs; however, the percentage decreased to only 3% in 2019 (latest data). This percentage was comparatively large in Greece during the economic and health crises, reaching 14.9% in 2014 and 12.1% in 2022 (Figure 2).

### 3.3. Greece’s Unmet Medical Health Needs by Region

**Figure 3:**
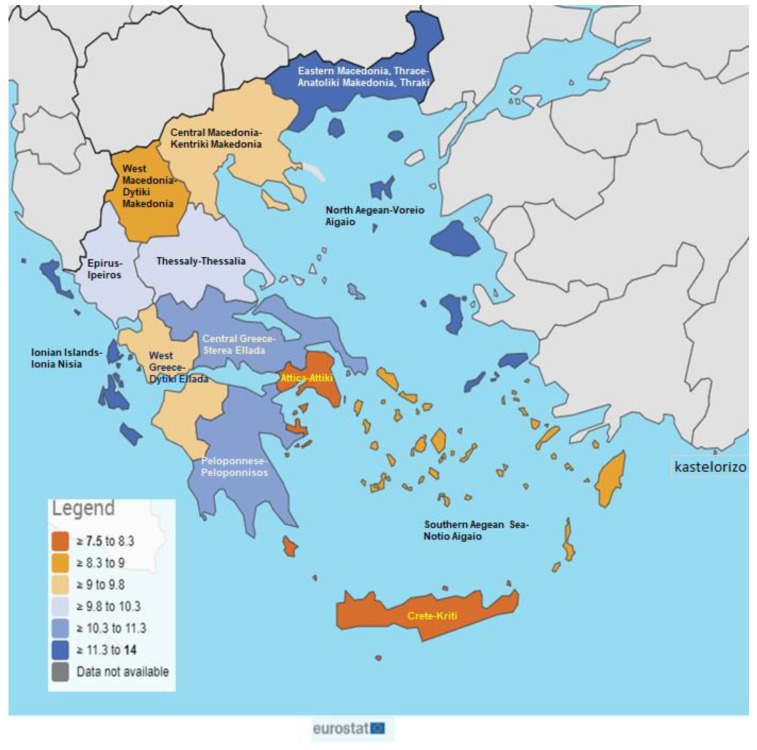
Geographical distribution of unmet health needs in Greece [26].

The Greek National Health System appears to have limitations in its ability to adequately cover the population, particularly in remote islands and mountainous regions of the country. There are a total of 269 hospital units distributed across the 13 regions of the country, with the majority (35%) situated in the Attica Region, followed by Central Macedonia (16%) in terms of their spatial distribution. The regions with the lowest percentage of hospitals are the Ionian Islands, Epirus, Northern Aegean, and South Aegean, collectively accounting for only 10% of the total hospital units in the entire country. Figure 3 presents the unmet health needs by region in Greece.

**Figure 4:**
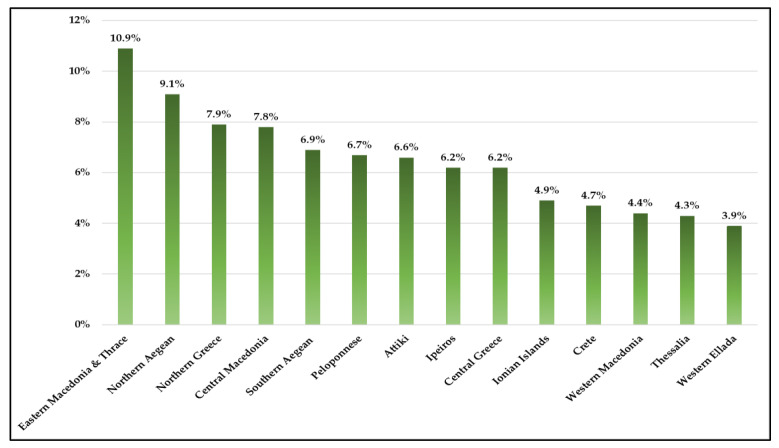
Unmet medical health needs by region for the year 2020 in Greece. Source: Eurostat 2022 (HLTH_SILC_08_R).

According to the data of Figure 4, throughout Eastern Macedonia and Thrace, approximately 10% of the population experienced unmet health needs. High percentages are shown for the Northern Aegean, Northern Greece, and Central Macedonia regions. Τhe percentage of unmet needs in Attica is interesting (6.6%), knowing that the majority of secondary and tertiary hospitals are located in this region. The regions with the lowest percentages of unmet needs (<5%) are the Ionian islands, Crete, Western Macedonia, Thessalia, and Western Greece.

### 3.4. Unmet Medical Health Needs in Greece versus EU27 by Income Quintile

**Figure 5:**
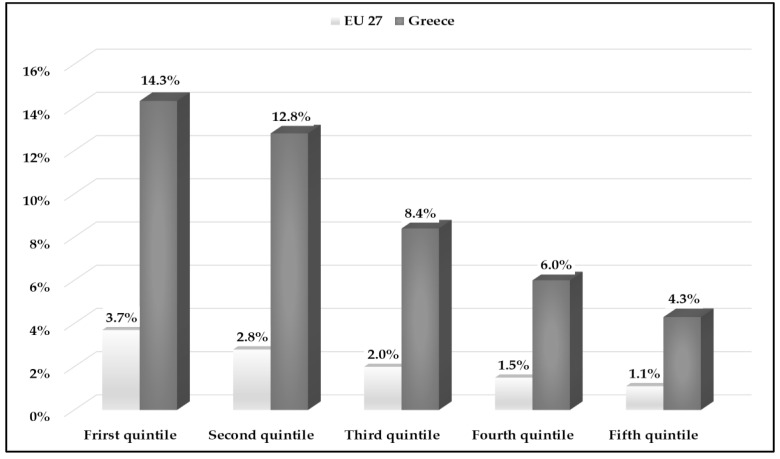
Unmet health needs for medical care by income quintile in Greece and EU27 (2022). Source: Eurostat: 2023 (HLTH_SILC_08).

The percentage of unmet needs declines as an individual’s income rises in the upper quintile. The first quintile encompasses 20% of the population with the lowest income. Conversely, the fifth quintile represents 20% of the population with the highest income. The first and second quintiles tended to have the greater percentages of unmet needs in comparison with the rest quintiles. Approximately 27% of the Greek population in the first and second quintiles stated unmet medical needs compared with 6.5% of the EU27 population (Figure 5).

### 3.5. Greece’s Unmet Medical Health Needs by Occupational Status

**Figure 6:**
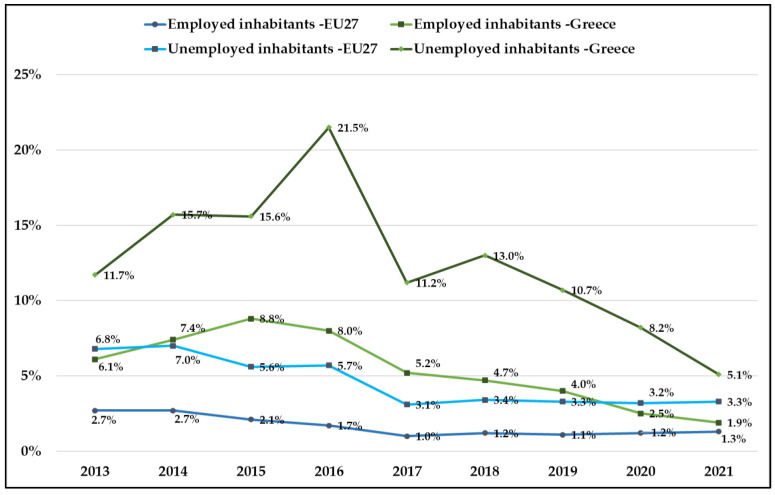
Employed and unemployed inhabitants’ unmet health needs in Greece and EU27. Source: Eurostat: 2022 (HLTH_SILC_13).

Regarding the distinction between employment and unemployment rates, it is important to mention that the rate of unemployment in Greece is approximately double in comparison to the other EU27 countries. However, it appears that, in Greece, during the recession and health crisis, approximately 12.5% of unemployed people and just 5% of employed people experienced unmet medical needs, in relation to EU27, where about 4.6% of unemployed and only 1.7% of employed people experienced unmet needs (Figure 6).

### 3.6. Comparative Effect Sizes of Key Variables Predictive of Unmet Needs

The analysis results, based on the Eurostat database, showed a significant positive correlation between unmet medical needs and bad/very bad self-perceived health (r = 0.786), some/severe self-perceived long-standing limitations in usual activities due to health problems (r = 0.681), and unemployment rate (r = 0.599), showing that an increase in bad/very bad health status, some/severe limitations, and unemployment correspond to a simultaneous increase in unmet medical needs (Table 2). On the contrary, unmet needs correlated negatively with private health expenditures (r = −0.682), public health expenditures (r = −0.765), very good/good health status (r = −0.793), real GDP per capita (r = −0.632), and employment rate (r = −0.615), showing that an increase in private and public health expenditures, very good/good health status, GDP per capita, and employment correspond to a simultaneous decrease in unmet medical needs (Table 2).

Table 3 presents the linear regression models regarding the predicted factors of unmet medical needs.

## 4. Discussion

To our knowledge, the present study is the first attempt to identify factors associated with self-perceived unmet needs during the economic and health crisis in Greece. The study, recording unmet needs in the health sector, is an essential indicator of how effectively the system has reached the wider social strata in terms of access to quality health services and in capturing the gap between the range of services required to satisfy different people’s needs and the services that are actually provided.

The results of this study showed that unmet health needs for medical care in Greece were twice as high as in the EU27, with consecutive increases during the economic and health crises. Similar were the findings regarding dental health needs, which indicated that these needs were almost three times higher in Greece compared to the EU27. Moreover, the unmet needs presented significant discrepancies among Greek regions, due to the morphological characteristics of the country (approximately 6000 large and small islands and remote areas) and the misallocation of health providers. The highest unmet needs were recorded in the Northern and Southern Aegean regions, as well as Eastern Macedonia, Thrace, and Northern Greece. Concerning income, our analysis displayed that a lower person’s income correlated with higher unmet health needs. Regarding occupational status, it has been proven that unemployed people are at a higher risk of experiencing unmet needs compared to employed individuals.

The result from the correlation and regression analyses showed that the odds of facing unmet health needs were higher for people with limitations in usual activities, those who report bad/very bad health status, unemployed, and people with low income. Low health expenditures (public or private) constituted significant predictive factors increasing the likelihood of unmet needs.

Our analysis indicated that approximately 1 in 10 citizens of Greece had unmet healthcare needs in the last decade, which is much higher in comparison to the other 27 European countries. During the economic crisis in Greece, it was observed that twice as many people experienced unmet health needs until 2016, when Law 4368/2016 provided the possibility for all people (regardless of insurance or not) to receive free-of-charge public health services, with the same copayment for the cost of medical care or drugs [27]. This decision led to an approximately 3% reduction in unmet healthcare needs within a year (2016–2017). However, during the pandemic the country mobilized healthcare resources to respond this urgent situation, causing a raise 3% within a year (2021–2022).

Where unmet health needs are concerned, dental care was one of the most serious. More than 10% of people in Greece reported unmet needs for dental care in the last decade, mainly for financial reasons. Public coverage for dental care costs is far more limited, due to restricted service packages and high levels of cost sharing [28,29]. On average, only one-third of total costs are borne covered by government schemes or compulsory insurance. In Greece, the level of compulsory coverage is very low; hence, consumers must pay significant out-of-pocket costs for dental care services, which are not part of the healthcare bundle [28].

Regional inequality of access to basic health services is significantly present in Greece. People living in remote regions and isolated islands encounter difficulties in reaching specialized medical practitioners and specialized laboratories [30]. As a result, citizens, particularly those facing health issues, are compelled to relocate to urban areas where suitable healthcare services are accessible. The main cause for this is that most hospitals, which are located in major urban centers, provide primary healthcare services. This is crucial, especially for people who need ongoing medical treatment. Consequently, the availability of healthcare depends on the individual’s place of residence and their proximity to appropriate facilities. This is consistent with our analysis that the region of Eastern Macedonia and Thrace had the highest frequency of unmet healthcare needs, as well as the islands of the Aegean. Surprisingly, despite Attica’s extensive concentration of hospitals, considerable unmet health needs were also observed. This also seems to be in line with the literature’s assertion that unmet health needs in home chronic care are affected by the lack of individualized, flexible health services and caregiver networks. Private providers and the one-dimensional perception of the patient’s treatment with medicine and prescription only lead to rising private health expenditures, filling the gap in primary healthcare services. A component of the Greek experience throughout the crisis appears to be linked to this conclusion [31].

A plethora of international studies have proven that the burden of unmet needs for healthcare falls mostly on people from low-income households [32,33,34]. This is in accordance with our results, whereby more than 13% of the low-income quintiles in Greece reported going without some medical care when they needed it in 2022 compared with only 1% among the high-income quintiles. Cost was the main reason for these unmet needs. Moreover, our analysis showed that increasing disposable income decreased the likelihood of unmet healthcare needs, which has been well documented in other countries with universal healthcare coverage [19,35,36]. In another similar study, it was mentioned that funding reduction in periods of crisis has a negative impact on access to healthcare, due to the increasing demand for services affecting waiting times, co-payments, and informal payments [37].

Furthermore, our analysis displayed that unemployment is a major risk factor of unmet medical needs. Although unemployed people in Greece have free access to healthcare services, problems regarding the use of healthcare have not been overcome [38,39]. The main reason was that unemployed people have to participate in the copayment of the cost of medicines or medical care in case they visit contracted physicians, despite very low income. Corresponding European studies agree with these results and pointed out that vulnerable groups, such as the unemployed, are more at risk of reduced access to health services, resulting in an increase in unmet needs and health inequalities [40,41]. It is a fact that health inequalities are associated with increased income inequality, which is a consequence of a political–economic process, such as unemployment [42]. During crises, health inequalities widen, with multiplying negative consequences for the unemployed population [43,44]. In line with previous studies, our model found that the probability of experiencing unmet healthcare needs was significantly increased for unemployed people.

Concerning health expenditures, after a period of low growth following the global financial crisis, annual per capita health expenditure growth picked up and reached 3% on average across EU member states between 2013 and 2019. However, in Greece, the growth rate was less than 1%, and the share of health spending financed through households’ out-of-pocket payments accounted for at least one-third of all health spending [6]. Our analysis indicated that an increase in public and private health expenditures led to a reduction in the population’s unmet health needs. Austerity measures in Greece led to a prolonged decline in the availability of healthcare services and financial security, as evidenced by a rise in unmet healthcare needs and significant expenses incurred due to health crises. According to a WHO report, low levels of public spending on health are associated with weak financial protection and high levels of unmet needs for health services [45]. Additionally, people with low incomes in Greece cannot afford to pay private health expenditures to fulfill their health needs, which are not covered by the public system; therefore, out-of-pocket payments create a financial barrier to access, resulting in an unmet need for healthcare.

Lastly, our results confirmed the claims from previous studies that the deterioration of the health status and the existence of functional limitations increase the likelihood of unmet needs. This finding coincides with the results of previous studies demonstrating that people claiming to be in good or very good health were less likely to perceive any unmet medical need [46,47,48]. Moreover, patients suffering from long-term health conditions frequently state that their healthcare needs remain unaddressed, and this situation has worsened with the increasing prevalence of chronic diseases [15,47]. However, it is worth mentioning the endogeneity between health status and unmet health needs. By focusing on contemporaneous values for unmet needs and health, it is difficult to evaluate the direction of causality between these two dimensions. Existing evidence suggests that past unmet health needs cause a worsening in present health status [48,49,50,51]. Furthermore, individuals who choose to forgo medical care may later encounter a decline in their overall health condition. Therefore, their health is likely to be severely affected if they do not receive timely and sufficient needed healthcare services; even worse, they are more likely to need expensive inpatient care (such as hospitalization), which will worsen their health. Because of this, associations between the aforementioned dimensions cannot be inferred to be causal, but bidirectional.

### Limitations of the Study

This study had some limitations that merit consideration. All data on unmet healthcare needs, health status, and limitations in usual activities due to health problems were based on self-perception; therefore, they were influenced, to some degree, by the respondents’ subjective perceptions and their social and cultural backgrounds. An additional limitation is that data were obtained from the EU-SILC, which do not cover the institutionalized population (individuals residing in health and social care institutions). This group tends to have a poorer health status compared to those living in private households. Consequently, it is probable that both data sources somewhat underestimated the proportion of the population with health issues. However, exclusion of these persons, who continuously receive healthcare services, might have resulted in an overestimation of unmet healthcare needs.

## 5. Conclusions

Access to healthcare services is reflected in the index of unmet health needs. The figures on Greek realities from Eurostat/EU-SILC demonstrate how challenging it is to increase access to healthcare. According to the study, Greece’s unmet health needs increased dramatically during the recession. They were additionally found to be higher in those who reported poor or extremely poor health, had limitations in daily activities, were unemployed, and had low income. Low public health spending has a negative impact on how easily people may get healthcare services. In addition to financial resources, service organization and distribution are essential. Accessibility issues are particularly delivered worse by a shortage of public health services, preventive care, or flexible primary care services that adapt to the needs of the user. However, access to health services is an important issue, but it also depends on a variety of elements, such as the severity of the disease, the socioeconomic status of the community as a whole, and the accessibility of a specific service. Many times, people go straight to the hospitals’ outpatient clinics, which typically offer primary healthcare, causing issues with service accessibility because of rising demand.

It Is suggested that, in order to improve the system, the role of interdisciplinary teams in the community must be further strengthened at the level of a local primary care network, focused on the health center, with the immediate recruitment of nurses and other medical specialists. It is important to enhance the health center’s capacity to record the health needs of the local population, as well as the function of the family doctor in regulating patient flows in hospitals. People’s reliance on primary healthcare services will increase as the health center becomes more prominent in the neighborhood. Conversely, the health center’s initiatives to promote health education as an opportunity to engage with the community and raise public knowledge of health-related concerns are seen as some of its strongest characteristics with potential for improvement.

It should be pointed out that health education includes not only the dissemination of information on health to particular population groups, but also the creation of incentives, capabilities, and self-care practices that are crucial for raising the level of health in the local community. It is possible to identify the underlying social, economic, and environmental issues, as well as other individual risk factors and behaviors, that have an impact on the community’s level of health through health education.

The effectiveness of incentives for improving the primary healthcare and strengthening the job performance of the personnel is also of the highest priority. It is essential to create a reliable set of quality indicators that will serve as the foundation for assessing the system’s performance. The procedures for collecting and employing information, as well as the static processing of outcomes, must be better supported. Supposing that home healthcare is established, patients, especially those with chronic diseases, will gradually be able make use of the services provided. To meet the needs of the people, it is important to finish the relevant institutional framework based on the use of the full range of scientific capabilities.

## Figures and Tables

**Table 1:** Description of indicators.

Indicators	Unit	Year	Reference	Description
Self-reported unmet need for medical care (too expensive or too far to travel or waiting list)	Percentage	2008–2022	TESPM110	Subjective view of a person’s need for examination or treatment, who did not receive it due to ‘financial reasons’, ‘waiting list’, or ‘too far to travel’
Gender (female)	Number	2013–2022	DEMO_PJAN__custom_6646580	
Gender (male)	Number	2013–2022	DEMO_PJAN__custom_6646561	
Household out-of-pocket payment (private health expenditures)	Million euro	2012–2021	HLTH_SHA11_HPHF__custom_6647166	Direct payment by households to health professionals, suppliers of pharmaceuticals, etc.
Government schemes and compulsory contributory healthcare financing schemes (public health expenditures)	Million euro	2012–2021	HLTH_SHA11_HPHF__custom_6647089	Government expenditures for the provision of health services
Self-perceived health	Percentage	2013–2022		One question instrument assessing the general perceived health: “How is your health in general? Is it very good/good/fair/bad/very bad?”
(Very good or good)			HLTH_SILC_01__custom_6646803	
(Fair)			HLTH_SILC_01__custom_6646814	
(Bad or very bad)			HLTH_SILC_01__custom_6646825	
People having a long-standing illness or health problem	Percentage	2013–2022	HLTH_SILC_04__custom_6647218	Chronic morbidity duration of at least six months
Self-perceived long-standing limitations in usual activities due to health problem (some or severe)	Percentage	2013–2022	HLTH_SILC_12$DEFAULTVIEW	One question from the Global Activity Limitation Instrument (GALI) assessing the presence of long-standing activity limitation
Real GDP per capita in EUR	Number	2008–2022	SDG_08_10	The average output of the economy per person measured in a base year
Employment rate	Percentage	2009–2022	TESEM010	The percentage of employed persons in relation to the total population
Unemployment rate	Percentage	2011–2022	TPS00203	The number of people unemployed as a percentage of the labor force

**Table 2:** Correlation analysis among unmet medical needs and predictors factors.

	Unmet Medical Needs	
	r	*p*-Value
Female	0.418	0.229
Male	0.280	0.433
Private health expenditures	−0.682 *	0.014
Public health expenditures	−0.765 **	0.004
Self-perceived health: fair	0.530	0.115
Self-perceived health: bad/very bad	0.786 **	0.007
Self-perceived health: very good/good	−0.793 **	0.006
Self-perceived long-standing limitations in usual activities due to health problem: some/severe	0.681 *	0.030
Real GDP per capita	−0.632 *	0.012
Employment rate	−0.615 *	0.019
Unemployment rate	0.599 *	0.040

** Correlation is significant at the 0.01 level (two-tailed); * correlation is significant at the 0.05 level (two-tailed).

**Table 3:** Simple linear regression models for unmet medical needs.

Adjusted R^2^	Predicted Factors	Dependent Variable	Unstandardized Coefficients		t	*p* Value	95.0% Confidence Interval for B	
			B	Std. Error			Lower Bound	Upper Bound
41.2%	Private health expenditures	(Constant)	31.921	7.860	4.061	0.002	14.408	49.435
		Unmet medical needs	−3.832	1.298	−2.953	0.014	−6.724	−0.941
54.4%	Public health expenditures	(Constant)	16.580	2.134	7.771	0.001	11.826	21.334
		Unmet medical needs	−0.754	0.201	−3.758	0.004	−1.201	−0.307
39.6%	Self-perceived long-standing limitations in usual activities due to health problem: some/severe	(Constant)	−34.598	16.760	−2.064	0.073	−73.247	4.050
		Unmet medical needs	1.859	0.708	2.627	0.030	0.227	3.491
57%	Self-perceived health: bad/very bad	(Constant)	0.833	2.430	0.343	0.741	−4.770	6.436
		Unmet medical needs	0.966	0.269	3.596	0.007	0.346	1.585
58.3%	Self-perceived health: very good/good	(Constant)	70.672	16.640	4.247	0.003	32.300	109.044
		Unmet medical needs	−0.807	0.219	−3.683	0.006	−1.313	−0.302
35.3%	Real GDP per capita	(Constant)	23.469	5.151	4.556	0.001	12.340	34.597
		Unmet medical needs	−0.001	0.000	−2.939	0.012	−0.001	0.001
32.6%	Employment rate	(Constant)	27.704	7.084	3.911	0.002	12.268	43.139
		Unmet medical needs	−0.325	0.120	−2.702	0.019	−0.586	−0.063
29.5%	Unemployment rate	(Constant)	3.720	2.345	1.586	0.144	−1.506	8.946
		Unmet medical needs	0.439	0.186	2.365	0.040	0.025	0.853

## Data Availability

Not applicable.
